# Severe asthma is really uncommon

**DOI:** 10.1186/2045-7022-3-S1-P18

**Published:** 2013-05-03

**Authors:** Anna Maria Zicari, Renato Cutrera, Valeria Lollobrigida, Ilaria Ernesti, Azzurra Cesoni Marcelli, Camilla Celani, Valentina De Vittori, Marzia Duse

**Affiliations:** 1 Sapienza Università di Roma, Policlinico Umberto I, Italy; 2 Broncopneumologia Ospedale Bambino Gesù, Roma, Italy; 3 Sapienza Università di Roma, Policlinico Umberto I, Immuno-Allergologia Pediatrica, Italy

## 

We describe the case of a 10-year-old girl with a history of severe persistent asthma and exercise-induced-asthma, controlled using an appropriate treatment with inhaled corticosteroid-long-acting beta-2 adrenergic agonists (ICS+LABA) and leukotriene receptor antagonists. She was healthy until the age of 8 years, when she presented two episodes of radiologically diagnosed pneumonia. After that, she began to present persistent cough, also nocturnal, stridor, dyspnea and respiratory distress and she was sent by pediatrician to our hospital. She performed a global spirometry which shows an obstructive and restrictive phenotype (FEV1: 75,3% and MEF50: 57,6%), without a significantly dilatation after inhaled salbutamol (400 mcg). She underwent to a systemic therapy with oral corticosteroid, with not benefit. She had no fever neither upper respiratory tract infections. We excluded gastro-esophageal reflux disease, cystic fibrosis, mycoplasma and chlamydia pneumonia. Cardiological examination was negative. During hospitalization, she spontaneously expectorated a thick fibrinous mucoid formation. A chest X-ray and a computed tomography (CT) scan showed atelectasis of both lung, widespread hyperlucency, and occlusion of the right main bronchus, compatible with a diagnosis of plastic bronchitis. Plastic bronchitis is a rare disease characterized by the formation of large gelatinous or rigid branching airway casts. The prevalence and etiology of plastic bronchitis are still unknown and the symptoms may also overlap with those of other diseases such as severe asthma, in the severe mucus plugging sometimes seen in allergic bronchopulmonary aspergillosis (ABPA) or in middle lobe syndrome. In the pathogenesis of the disease the inflammation is usually present and initiates cast formation. Treatment includes bronchodilators, inhaled and oral corticosteroids, mucolytics, airway clearance therapy and antibiotics. Other therapies can include inhaled heparin, urokinase, tissue plasminogen activator (TPA), dornase alfa and oral macrolide antibiotics as mucoregulatory therapy 2.

## Conclusions

The presence of asthmatic symptoms without clinical improvement after appropriate therapy is not always suggestive of severe asthma. Therefore, for the appropriate diagnosis, we have to exclude the other lung diseases and, among the differential diagnoses, is possible to consider also plastic bronchitis.

